# Taking off the gown: Impact of discontinuing contact precautions for extended-spectrum β-lactamase (ESBL)–producing organisms

**DOI:** 10.1017/ash.2021.189

**Published:** 2021-10-21

**Authors:** Lindsey B. Gottlieb, Emily Walits, Gopi Patel, Sarah Schaefer

**Affiliations:** 1 Division of Infectious Diseases, Department of Medicine, Icahn School of Medicine at Mount Sinai, New York, New York; 2 Department of Infection Prevention, The Mount Sinai Hospital, New York, New York

## Abstract

Contact precautions are used to prevent the spread of extended-spectrum β-lactamase (ESBL)–producing organisms in acute-care hospitals, but supporting data are lacking. We discontinued such precautions for ESBL *Escherichia coli* and *Klebsiella* spp and found no increased prevalence of these organisms with our change in practice.

Infection control guidelines recommend contact precautions for extended-spectrum ß-lactamase (ESBL)–producing organisms in the acute-care setting.^1,2^ However, limited data support this practice, and recent studies have challenged the assumption that contact precautions reduce the spread of such organisms.^3–6^ Our institution discontinued the use of contact precautions for ESBL in September 2017. Herein, we describe the impact of this change on our burden of ESBL organisms.

## Methods

### Study setting

The Mount Sinai Hospital is a 1,018 bed, tertiary-care, academic medical center in New York City. Excluding intensive care units and obstetrics, which have predominantly single-occupant rooms, there are 644 beds in the adult, acute-care hospital, of which only 225 (35%) are in single-occupant rooms. Until September 13, 2017, contact precautions were implemented for all patients colonized or infected with an ESBL organism. Contact precautions in our institution include the use of single-occupant rooms and require all who enter to wear gown and gloves. In mid-September 2017, contact precautions were discontinued for all ESBL-positive patients. Concurrently, precautions for methicillin-resistant *Staphylococcus aureus* (MRSA) and vancomycin-resistant *Enterococcus* (VRE) were relaxed to only require contact precautions for active MRSA infections, as opposed to colonization, and only for daptomycin- or lineolid-resistant VRE. In November 2014, our institution introduced the Joint Commission’s Targeted Solutions Tool to improve hand hygiene compliance throughout the hospital.^7^ The Targeted Solutions Tool helps institutions identify and address barriers to hand hygiene compliance. Compliance is monitored through direct, covert observations from individuals normative to the units. All inpatient units had reached the “improve” phase by January 2017, meaning they had completed baseline hand hygiene observations and were actively implementing processes to improve their compliance.

### Study design

We performed a retrospective, observational, quasi-experimental study comparing overall patient prevalence rates of ESBL *Escherichia coli* and ESBL *Klebsiella* spp between the 11-month periods leading up to and following the change in practice (October 2016–August 2017 and October 2017–August 2018). Positive cultures were identified using VigiLanz software (VigiLanz, Minneapolis, MN). Although active surveillance cultures to detect ESBL colonization were not routinely performed during this period, all positive cultures were included in the analysis, regardless of whether they were considered clinically significant. Overall patient prevalence rates were determined using National Healthcare Safety Network (NHSN) laboratory-identified definitions and compared between the periods.^8^ Emergency department (ED) prevalence rates were also calculated as a proxy for our catchment area and were compared to detect any difference in our baseline ESBL rates. ESBL isolates were defined as those that screened positive by the VITEK 2 system (bioMérieux, Marcy l’Etaile, France). Clinical and Laboratory Standards Institute 2014 break points were used. We included adult patients in our acute care hospital from whom clinical cultures had been taken. Isolates identified as carbapenem-resistant Enterobacterales (CRE) were excluded. Isolation gown costs were calculated based on materials purchasing.

### Statistics

Prevalence rates were compared using Poisson regression. Analyses were performed using SAS version 9.4 software (SAS Institute, Cary, NC). Hand hygiene rates were compared using a 2-proportion *Z* test.

## Results

We detected no change in the prevalence of inpatient or ED ESBL *E. coli* or *Klebsiella* spp after discontinuing contact precautions (Table 1). We also detected no change in the prevalence of either organism from inpatient cultures collected after hospital day 3 (Table 2). Hand hygiene compliance improved from 84% to 89% between study periods (*P* < .0001). Isolation gown utilization decreased by 23% from 1,619,710 units in October 2016–August 2017 to 1,246,315 units in October 2017–August 2018. This change corresponded to a 24% reduction in spending, for a cost avoidance of $121,441.

**Table 1.** Prevalence Rates of ESBL Organisms Before and After Discontinuation of Contact Precautions (CP)

Organism	Rate Pre-CP Discontinuation (Cultures/100 Admissions)	Rate Post-CP Discontinuation (Cultures/100 Admissions)	Rate Ratio (95% CI)	*P* Value
*Escherichia coli* (inpatient)	0.65 (342/523.06)	0.65 (311/478.77)	0.99 (0.85–1.16)	.93
*Klebsiella* spp (inpatient)	0.31 (160/523.06)	0.33 (159/478.77)	1.09 (0.87–1.25)	.46
*Escherichia coli* (ED)	0.20 (205/1,017.86)	0.22 (224/1,041.03)	1.07 (0.88–1.29)	.49
*Klebsiella* spp (ED)	0.06 (66/1,017.86)	0.07 (77/1,041.03)	1.14 (0.82–1.58)	.43

Note. ESBL, extended-spectrum β-lactamase; CI, confidence interval; ED, emergency department.

**Table 2.** Prevalence Rates of ESBL Organisms Collected After Hospital Day 3

Organism	Rate pre-CP Discontinuation (Cultures/100 Admissions)	Rate post-CP Discontinuation (Cultures/100 Admissions)	Rate Ratio (95% CI)	*P* Value
*Escherichia coli* (inpatient)	0.24 (128/523.06)	0.22 (105/478.77)	0.90 (0.69–1.16)	.41
*Klebsiella* spp (inpatient)	0.15 (78/523.06)	0.14 (66/478.77)	0.92 (0.66–1.28)	.64

Note. ESBL, extended-spectrum β-lactamase; CP, contact precautions; CI, confidence interval; ED, emergency department.

## Discussion

When deciding whether to implement contact precautions for a given organism, institutions must consider the endemicity of the organism in its catchment area, the ability of that organism to transmit between patients, and the effectiveness of contact precautions in preventing such transmission. To inform this process, infection preventionists and healthcare epidemiologists refer to consensus and evidence-based guidelines. Unfortunately, the limited guidelines specific to ESBL organisms are problematic.

In 2006, the CDC recommended contact precautions for all patients colonized or infected with multidrug-resistant organisms, irrespective of genus or species. Notably, none of the literature cited for this recommendation relates to ESBL organisms.^1^ The European Society of Clinical Microbiology and Infectious Diseases also recommends contact precautions for ESBL Enterobacterales. However, referenced studies evaluated contact precautions as part of larger infection prevention bundles or were undertaken in an outbreak setting; thus, conclusions about the effectiveness of contact precautions alone based on these data must be made with caution.^2^ Although the Society of Healthcare Epidemiology of America more recently released guidelines describing when to discontinue contact precautions, they did not define when to initiate such precautions.^9^

In fact, literature exists to dispute the hypothesis that ESBL organisms spread rapidly between inpatients. Several studies have shown low rates of cross transmission of ESBL organisms from index to contact patient.^4–6^ In some cases, suspected transmissions were disproven using whole-genome sequencing.^4,6^ One potential explanation is that ESBL organisms, particularly ESBL *E. coli*, are not a frequent cause of environmental contamination.^5^ Equally as important, contact precautions have not been shown to independently reduce the nosocomial spread of these organisms in the non-outbreak setting.^3,4^

Ultimately, we felt that the evidence was insufficient to support the continued use of contact precautions for ESBL organisms given the well-described negative impacts of contact precautions for both the patient and the institution.^10^ Our decision was also influenced by our need to reserve our limited number of single-occupant rooms for emerging, highly transmissible organisms, most notably CRE and *Candida auris*.

Our study had several limitations. First, it was retrospective, raising the possibility of confounders. However, we found no difference in our ED ESBL prevalence rates between the study periods, suggesting that our community rates remained stable throughout. Although we detected a small but statistically significant improvement in hand hygiene compliance between study periods, this finding only underscores the importance of focusing on the basic tenets of infection prevention.

We calculated patient prevalence rates rather than infection/colonization incidence rates, which are a more direct measure of healthcare acquisition according to the NHSN.^8^ A significant proportion of our patients seek care at multiple health systems; thus, we could not reliably capture prior ESBL infection or colonization to calculate incidence rates. Instead, we compared ESBL prevalence rates between cultures collected after hospital day 3, as an indicator of hospital acquisition, and we did not detect a difference between study periods. Lastly, our findings were consistent for both ESBL *E. coli* and *Klebsiella* spp, but our absolute number of *Klebsiella* isolates was relatively small. This finding underscores the need for additional research evaluating the impact of contact precautions on the transmissibility of non–*E. coli* ESBL spp.

In conclusion, discontinuing contact precautions was not associated with an increase in the prevalence of ESBL *E. coli* or *Klebsiella* spp in our hospital. As a result of our change in practice with respect to contact precautions for ESBL, MRSA, and VRE, we also generated a cost avoidance on materials. The coronavirus disease 2019 pandemic has only highlighted the significance of our study, underscoring the need to reserve single-occupant rooms and personal protective equipment for emerging highly infectious pathogens. Our findings add to the growing body of literature questioning the need for such precautions for ESBL organisms, particularly in institutions similar to ours, with limited availability of single-occupant rooms and prevalence of ESBL in our catchment area.
